# Investigating habitat preferences and host-feeding behavior of mosquito disease vectors on commercial swine farms in the USA

**DOI:** 10.1186/s13071-026-07349-6

**Published:** 2026-03-12

**Authors:** Stephen Edache, David Edache, Lauryn Mauler, Ashley Thackrah, Vanessa Horton, Christy Hanthorn, Andrea L. Dixon, Cassandra Olds, Dustin Swanson, Dana Mitzel, Chad Mire, Lee W. Cohnstaedt, Natalia Cernicchiaro

**Affiliations:** 1 Center for Outcomes Research and Epidemiology, Department of Diagnostic Medicine and Pathobiology, College of Veterinary Medicine, Kansas State University, Manhattan, KS USA; 2 Department of Entomology, College of Agriculture, Kansas State University, Manhattan, KS USA; 3 Foreign Arthropod-Borne Animal Diseases Research Unit, Agricultural Research Services, United States Department of Agriculture, National Bio and Agro-Defense Facility, Manhattan, KS USA

**Keywords:** Arbovirus, Blood meal, Feeding behavior, Habitat preference, Host, Mosquito, Swine farms, Vector

## Abstract

**Background:**

Mosquito feeding behavior and habitat preferences strongly influence arbovirus transmission, particularly for viruses where swine serve as amplifying hosts. The high density of pigs in commercial operations poses a potential risk for severe outbreaks if mosquito-borne pathogens were to be introduced. This study characterizes habitat preferences and feeding behavior of mosquito species on commercial swine farms in the USA.

**Methods:**

Overall, ten farms (five sow and five wean-to-market [WTM] farms) were sampled biweekly from June to October 2024. At each farm, resting mosquitoes were collected outdoors using aspirators, while light traps were deployed indoors and outdoors to trap host-seeking mosquitoes. Blood-fed females from all capture methods underwent DNA extraction, polymerase chain reaction (PCR), and Sanger sequencing to identify mosquito species and vertebrate hosts. Generalized linear mixed models were used to evaluate associations between habitat, location, and calendar period with the abundance of resting and blood-fed mosquitoes.

**Results:**

In this study, 3009 resting mosquitoes (both males and females) were collected. Vegetation habitats consistently harbored more resting and male mosquitoes than water bodies (*P* ≤ 0.02), on both sow and WTM farms. A total of 444 blood-fed female mosquitoes were captured, of which 25.7% (114/444) were captured on sow farms and 74.3% (330/444) on WTM farms. On WTM farms, the abundance of blood-fed mosquitoes was significantly higher indoors than outdoors (*P* < 0.01). Across production systems, the dominant blood-fed species were *Anopheles quadrimaculatus* (*n* = 157), *Culex pipiens* (*n* = 105), *Aedes vexans* (*n* = 65), *Culex salinarius* (*n* = 46), and *Anopheles punctipennis* (*n* = 45). Pigs accounted for the majority of blood meals (*n* = 384), followed by white-tailed deer (*n* = 38), cattle (*n* = 11), and birds (*n *= 11). The probability of mosquitoes feeding on pigs compared with other hosts was significantly higher from late June through mid-September (*P* < 0.01).

**Conclusions:**

This study provides baseline data on mosquito habitat preferences and feeding behavior on US commercial swine farms. Routine mosquito vector surveillance and targeted control measures, both indoors and in surrounding habitats, should be integrated into herd biosecurity programs to mitigate mosquito-associated health risks in commercial swine operations.

**Graphical Abstract:**

**Figure:**
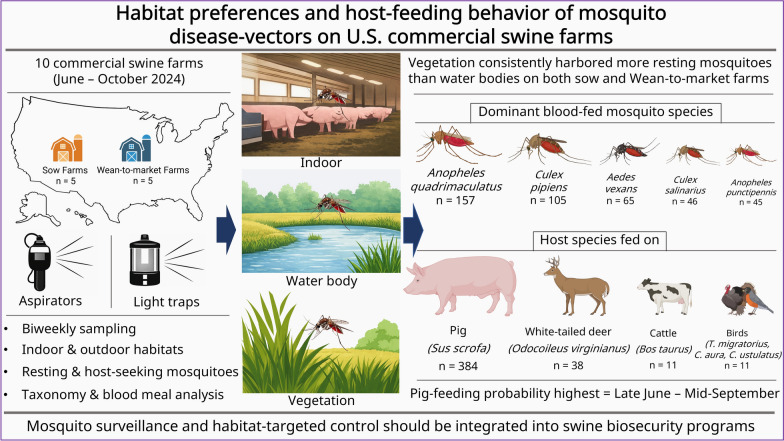

**Supplementary Information:**

The online version contains supplementary material available at 10.1186/s13071-026-07349-6.

## Background

Several arboviruses of veterinary and public health concern, including but not limited to West Nile virus (WNV), Japanese encephalitis virus (JEV), Saint Louis encephalitis virus (SLEV), and Rift Valley fever virus (RVFV), are maintained and facilitated through the complicated relationship between mosquitoes, their hosts, and the dynamics of mosquito blood-feeding behavior [1]. Pathogen transmission requires that a mosquito acquire a pathogen during a blood meal from a vertebrate host and subsequently feed on a susceptible recipient host. Thus, feeding patterns are a critical determinant of vector competence and transmission dynamics [2].

Mosquito species display diverse blood-feeding behaviors, ranging from strict human preference to opportunistic feeding on both humans and animals [1]. Host choice and feeding success are shaped by a complex interplay of factors, including the availability, accessibility, population size, population density, and defensive behaviors of the host; proximity to mosquito habitats; and environmental conditions [2–6]. Intrinsic mosquito traits (e.g., genetics, nutritional state, and reproductive status), extrinsic factors (e.g., host abundance and kairomones), and climatic variables further influence feeding outcomes and pathogen transmission potential [7–10]. Since blood meals provide proteins and lipids essential for egg development and reproductive fitness, host choice remains a central determinant of mosquito biology and vectorial capacity [11].

Following a blood meal, female mosquitoes typically seek resting sites to digest the ingested blood and complete egg development [12]. Such sites are typically shaded, humid, and cooler than the surrounding environment, offering protection from desiccation, temperature extremes, and wind [12]. By reducing physiological stress, these microhabitats increase mosquito survival, prolong adult longevity, and allow sufficient time for blood meal digestion and egg development [13–15]. In turn, the availability of these suitable resting sites can directly influence vectorial capacity, since longer-lived females have a higher probability of surviving the extrinsic incubation period of arboviruses and transmitting infection to subsequent hosts [16].

Studies conducted outside the USA have shown that host-seeking mosquitoes can enter pig barns, feed on pigs [17, 18], survive indoors for extended periods, and adjust their feeding patterns to the most abundant mosquito species and hosts present [18]. These observations raise important considerations: Feeding preferences among mosquito species may be shaped by ecological factors and host abundance, and certain indoor and/or outdoor habitats or daily farm activities may create favorable conditions for mosquito breeding. Addressing these concerns is crucial for developing comprehensive mosquito management strategies on pig farming operations.

While mosquito feeding behavior and preferred habitats have been examined in various ecological contexts, including livestock farms [19], little is known about their feeding patterns and resting habitat preferences on US commercial swine farms. This is a critical knowledge gap because pigs serve as amplifying hosts for some medically important arboviruses and are raised under intensive production systems characterized by high animal density, enclosed barn structures, and stringent biosecurity practices designed to limit the spread of infectious pathogens. Such conditions may influence mosquito access, resting behavior, and feeding opportunities, and the introduction of mosquito-borne pathogens into these high-density host environments could trigger severe outbreaks with significant animal health and economic consequences. Therefore, the objective of this study was to characterize the habitat preferences and feeding behavior of mosquito disease vectors on commercial swine farms in the USA, providing baseline data to inform vector surveillance and biosecurity strategies in swine production systems.

## Methods

A detailed description of the study area, swine farms, sampling period, sampling procedure, and specimen identification methods has been published previously [20]. Briefly, this study was conducted on commercial swine farms in southeast Iowa, the top hog-producing state in the US. Farms were eligible for study inclusion if they had visible mosquito-rich habitats (pond or lagoon, crop field, or vegetation), had management that allowed indoor sampling, and were within our designated travel area. A total of ten farms were enrolled—five sow and five wean-to-market (WTM) farms—representing a range of biosecurity protocols and ventilation systems. However, because some farms withdrew and were replaced mid-season, no more than four farms were sampled at any individual visit for each production system. Sow farms housed an average of 2500 sows on slatted floors under high biosecurity measures, and barns were equipped with mechanically powered ventilation systems, whereas WTM farms had comparable herd capacities but intermediate biosecurity measures were in place, and the barns used a variety of ventilation systems, such as natural, tunnel, and a combination of curtains and fans.

### Sampling schedule

Sample collections took place over ten visits from June to October 2024. Sampling occurred every 2 weeks, with two consecutive collection days per visit. Mosquito traps were deployed overnight between 1700 and 0600; traps were set up on the first evening of each sampling visit and the captured specimens retrieved the following morning. Later that same day, traps were reset, and capture bags retrieved the next morning. For clarity and ease of interpretation, each sampling visit was grouped into distinct calendar periods on the basis of the sampling dates, as depicted in Supplementary Table 1.

By design, one BG-Pro (BG) light trap (Biogents AG, Regensburg, Germany), configured in the Centers for Disease Control and Prevention (CDC)-style setup, was placed indoors on each farm near visible cobwebs or dark corners of the barn. A single indoor trap was used to minimize repeated handling and movement of equipment within barns, particularly across production phases on sow farms, to reduce the risk of breaching farm biosecurity protocols. On sow farms, indoor traps were strategically placed within gestation barns, given the known susceptibility of pregnant sows to arbovirus-associated reproductive losses. Two additional traps were positioned outdoors—one near water bodies (pond or lagoon) and one in surrounding vegetation or crop fields. Sewage lagoons consisted of open anaerobic systems receiving manure and wastewater from barns, whereas ponds were characterized by stagnant water associated with runoff management or natural depressions. Vegetated habitats included crop-adjacent areas, fallow fields, and natural vegetation zones dominated by shrubs, grasses, and native trees. The average distance between farms within each production system was 7.6 km for sow farms and 20.8 km for WTM farms (Supplementary Table 2). Across all farms, the average distance between indoor traps and traps placed near water bodies was 149.6 m, between indoor traps and vegetation traps was 268.7 m, and between vegetation traps and water-body traps was 244.4 m (Supplementary Table 3). Indoor traps used ultraviolet (UV) light as a visual attractant and were operated without CO_2_. Outdoor traps were baited with dry ice to slowly dispense CO_2_ to attract host-seeking mosquito vectors and were operated without UV light.

To assess the abundance of adult resting and engorged female mosquitoes, we used battery-powered aspirators for 10 min at each designated outdoor environment. Because nocturnal mosquitoes typically seek resting sites at sunrise, aspirations were performed before 0600 on the day of sample collection.

### Specimen handling and identification

Specimens from BG traps were immediately anesthetized using dry ice, while specimens obtained using aspirators were anesthetized using triethylamine (FlyNap^®^, Carolina Biological Supply Company, Burlington, NC, USA) soaked in a cotton ball within a sealed collection jar. All specimens were transferred into Falcon tubes, preserved on dry ice, and transported to the entomology laboratory at Kansas State University and stored at −80 °C until processing. Following sorting of the specimens, mosquitoes were sorted by species and sex, then counted. Species or species group identification used the IDX system (Vectech Inc., Baltimore, MD, USA), followed by expert verification by trained entomologists. Samples were subsequently stored in vials at −80 °C until further analysis. Closely related species (e.g., *Culex pipiens*/*restuans*) were grouped when morphological separation was not feasible. Damaged specimens lacking key morphological features necessary for identification were classified to genus level or recorded as “unknown.”

### Blood meal extraction

The blood meal extraction was performed using previously established procedures [21]. Briefly, blood-fed female mosquitoes were separated from other specimens under a stereomicroscope on the basis of the presence of visible abdominal blood. Using sterile pipette tips, the abdominal contents were expelled onto the sampling area of QIAcard FTA^®^ Classic Cards (QIAGEN, Hilden, Germany) by gently rolling from the thorax–abdomen junction toward the tip of the abdomen. Blood was smeared into a compact circular area, avoiding contact with the head, thorax, wings, and legs to minimize contamination. Specimens were individually labeled, and the degree of blood meal digestion was visually estimated on a three-point scale (fresh, partially digested, or well digested) [21]. Cards were air-dried for ~10 min and stored in zip-sealed plastic bags within boxes at ambient temperature until DNA extraction. This method preserves host DNA (and potentially pathogen DNA/RNA) at room temperature for extended periods, enabling downstream polymerase chain reaction (PCR)-based blood meal analysis without the need for cold-chain storage.

### DNA extraction from blood meals and mosquitoes

As previously detailed [22], DNA was extracted from mosquito blood meals preserved on QIAcard FTA^®^ Classic Cards using a modified HotSHOT (hot sodium hydroxide and Tris) method. Briefly, two 1-mm punches from each preserved blood meal were placed into individual wells of a 96-well PCR plate. Each well received 50 µL of lysis solution (25 mM NaOH, 0.2 mM EDTA, pH 12) and was incubated at 95 °C for 60 min, followed by cooling to 4 °C. After brief centrifugation, 50 µL of neutralization solution (40 mM Tris–HCl, pH 5) was added to each well. Plates were vortexed, centrifuged, and stored at −20 °C or −80 °C until PCR amplification. This approach was selected as it allows rapid, low-cost extraction of host DNA from preserved blood meals, with minimal sample consumption, so that the remaining material on the card can be archived for future analyses.

DNA was extracted from individual mosquitoes using the ZymoBIOMICS DNA Miniprep Kit (Zymo Research, USA) following the manufacturer’s instructions. Each specimen (≤ 15 mg) was placed into a ZR BashingBead^™^ Lysis Tube with 750 µL of ZymoBIOMICS™ Lysis Solution and homogenized in a Fisherbrand^™^ 4 mL Mini Bead Mill Homogenizer (Fisher Scientific) at maximum speed for ~30 s. Lysates were centrifuged (≥ 10,000*g*, 1 min), and up to 400 µL of supernatant was passed through a Zymo-Spin^™^ III-F Filter. DNA was bound by adding 1200 µL of DNA binding buffer, applied to a Zymo-Spin^™^ IICR Column, and washed sequentially with DNA wash buffer 1 (400 µL) and DNA wash buffer 2 (700 µL, then 200 µL). DNA was eluted in 100 µL DNase/RNase-free water, filtered through a Zymo-Spin^™^ III-HRC Filter to remove PCR inhibitors, and stored at −20 °C pending downstream analysis.

### PCR amplification for blood meals and mosquito DNA

Amplification of the extracted DNA was performed using established protocols [23]. Briefly, DNA extracted from mosquito blood meals preserved on FTA cards was amplified using a three-step, hierarchical PCR (A → B → C) designed for progressively shorter amplicons, targeting the vertebrate mitochondrial cytochrome c oxidase I (*COI*) gene (Supplementary Table 4). For the extracted mosquito DNA, a fragment of the mitochondrial cytochrome c oxidase subunit I (*COI*) gene was amplified using universal barcoding primers (Supplementary Table 4).

Negative (no-template) controls were included in each PCR run to monitor for contamination. Amplicons were visualized on 1.5% agarose gels stained with ethidium bromide, and bands of the expected size were submitted to Eurofins Genomics DNA sequencing laboratory in Louisville, KY, for PCR product clean-up and Sanger sequencing. Sequencing was performed using the forward primer only; if initial results were poor or unsuccessful, the corresponding reverse primer was used. Resulting chromatograms were trimmed, quality checked, and compared against the National Center for Biotechnology Information (NCBI) GenBank database using Basic Local Alignment Search Tool (BLAST), with a threshold of ≥ 98% sequence identity for host and mosquito species assignment. Sequences with < 98% identity were resolved by comparison with known local host communities and geographical distributions of vertebrate species [23].

### Data analysis

Descriptive and inferential statistical analyses were conducted to evaluate mosquito resting habitat preferences, blood-fed mosquito abundance, and mosquito feeding patterns across sow and WTM farms. Definitions of all outcomes and explanatory variables are provided in Supplementary Table 1. Data on overall mosquito abundance and species diversity based on BG trap collections have been published elsewhere [20].

Descriptive statistics included totals, means, and ranges of the number of resting mosquitoes; percentage of male mosquitoes; and the proportion and percentage of blood-fed female mosquitoes. These summaries were stratified by calendar period (early June to early October), location (indoors and outdoors [water bodies, vegetation]), and production system (sow and WTM farms). The number of host blood meals was summarized by mosquito species, location, capture method (trap and aspirator), and production system.

Generalized linear mixed models (GLMMs) were fitted using a residual pseudo-likelihood method with Newton–Raphson with ridging optimization and Kenward–Roger degrees of freedom adjustment. Each model was initially fitted using the Laplace method to evaluate for overdispersion by assessing the Pearson *χ*^2^/*df* statistic [24]. Repeated measures were modeled using several alternative covariance structures (including spatial power, autoregressive, and unstructured), and random slope models were also evaluated. These models were compared using Akaike’s information criterion (AIC) and the Bayesian information criterion (BIC), with lower values indicating better fit. The covariance structure that produced the best balance of model fit and parsimony on the basis of these information criteria was selected for the final analysis. Separate models were fit for sow and WTM farms.

To estimate mosquito abundance (including both males and females) from aspirator collections near water bodies and in vegetation habitats, a GLMM with a Poisson distribution and log link function was fitted and included fixed effects of calendar period, habitat, and their two-way interaction, with random intercepts for farm and a spatial power covariance matrix for calendar period to account for repeated sampling of trap ID nested within farm. If the two-way interaction was not significant (*P* > 0.05), the interaction was removed and the model re-fitted with only the main effects. Early and late June data for WTM farms were excluded owing to estimation issues encountered in the model as a result of low counts and limited farm representation.

To estimate the proportion of male mosquitoes (number of male mosquitoes divided by the total number of mosquitoes [male + female]) captured using aspirators from each habitat, a GLMM with a binomial distribution and logit link function was fitted and included fixed effects of calendar period, habitat, and their two-way interaction, and random intercepts for trap ID nested within farm. If the two-way interaction was not significant (*P* > 0.05), the interaction was removed and the model re-fitted with only the main effects. For WTM farms, data from the early and late June, mid-August, late September, and early October calendar periods were excluded from the analysis owing to estimation issues caused by the absence of male mosquitoes near water bodies.

To estimate the abundance of blood-fed female mosquitoes trapped using BG traps by trap location, a GLMM with a Poisson distribution and log link function was fitted and included calendar period and location as fixed effects, with random intercepts for farm and a first-order autoregressive covariance structure to account for repeated measures within each farm. To prevent model overfitting, calendar periods were aggregated into monthly intervals (“calendar months”). Owing to estimation issues, calendar months with no blood-fed mosquito captures, such as October for sow farms and June for WTM farms, were excluded from the analysis.

To estimate the probability of mosquito species (captured using BG traps and/or aspirators) feeding on pigs versus other hosts, a GLMM with a binary distribution and logit link function was fitted and included fixed effects of mosquito species, calendar period, and location (only for WTM farms), and a random intercept for farm. To address sparse data, some mosquito species were grouped into broader categories (Supplementary Table 1). Owing to estimation issues, calendar periods with absent or very few blood-fed mosquito captures in mid and late September and early October for sow farms, and early/late June, early/late July, and early October for WTM farms, were excluded from the analysis.

Model-adjusted means, 95% confidence intervals, and *P*-values were obtained from the mixed-effects models outputs. Post hoc comparisons were adjusted using the Tukey method to control for multiple testing. Significant difference was set at *P* < 0.05. All descriptive statistics, tables, and figures were generated using the R language [25], and GLMMs were implemented in the SAS software (version 9.4; SAS Institute Inc., Cary, NC, USA) using Proc Glimmix.

## Results

### Abundance of adult resting mosquitoes

A total of 3009 resting mosquitoes (males and females) were collected using aspirators across sow and WTM farms between June and October 2024. Of these, 66.8% (2011/3009) were collected on sow farms, and 33.2% (998/3009) on WTM farms. On sow farms, 43.4% (872/2011) and 56.6% (1139/2011) of the mosquitoes were collected near water bodies and vegetation, respectively, whereas on WTM farms, 14.3% (143/998) and 85.7% (855/998) of the mosquitoes were collected near water bodies and vegetation habitats, respectively.

### Habitat-specific abundance of resting mosquitoes

**Fig. 1:**
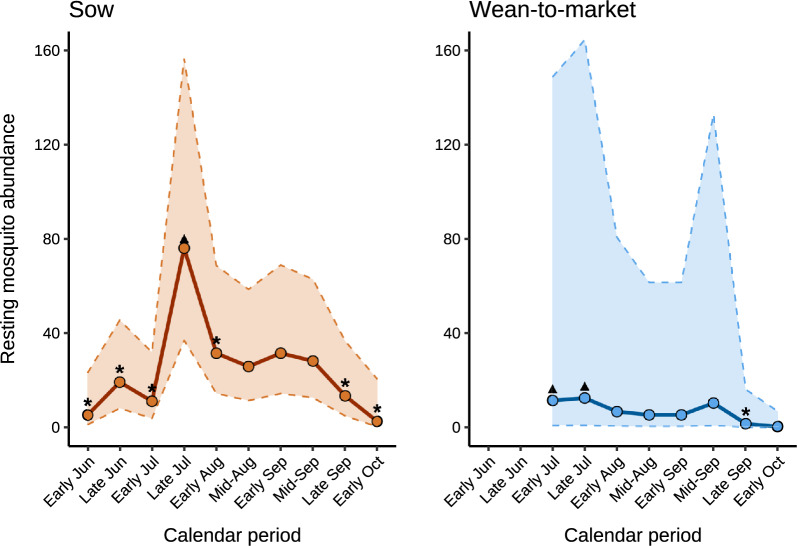
Model-adjusted mean counts of resting mosquitoes by calendar period on sow and wean-to-market farms. Circles (o) indicate model-adjusted mean counts of resting mosquitoes (males and females) collected with aspirators across habitat (water body and vegetation) and individual farms during each calendar period. Dashed lines (–) represent the 95% confidence intervals. Triangles (▲) indicate periods with significantly higher estimates than those marked with asterisks (*). Significant contrasts for sow farms were late July versus early and late June, early July, early August, late September, and early October (*P* < 0.05). Significant contrasts for wean-to-market farms were late September versus early and late June (*P* < 0.05). Early and late June data for wean-to-market farms were excluded owing to estimation issues encountered in the model as a result of low counts and limited farm representation

The total, mean, and range of resting mosquitoes collected using aspirators near water bodies and vegetation for each calendar period, across farms within each production system, are presented in Supplementary Tables 5 and 6. The interaction of habitat and calendar period on resting mosquito abundance was not statistically significant for sow (*P* = 0.67) and WTM (*P* = 0.50) farms. After dropping the interaction and evaluating the main effects, for sow farms, there was no significant difference between habitat type (*P* = 0.12). The model-adjusted estimates of mean mosquito counts were 13.4 (95% confidence interval [CI] 6.3–28.6) near water bodies and 21.8 (95% CI 10.6–44.7) in vegetation on sow farms. Resting mosquito counts varied significantly by calendar period on sow farms (*P* < 0.01), with a higher count of resting mosquitoes in late July compared with early and late June, early July, early August, late September, and early October (Fig. 1). On WTM farms, resting mosquito counts significantly varied between habitat types (*P* < 0.01); the mean count of resting mosquitoes was 1.9 (95% CI 0.2–22.2) near water bodies and 10.7 (95% CI 0.8–149.0) in vegetation. Additionally, resting mosquito counts varied significantly by calendar period (*P* < 0.01), with lower counts observed in late September compared with those in early and late July (Fig. 1).

### Habitat-specific proportion of male mosquitoes

**Fig. 2:**
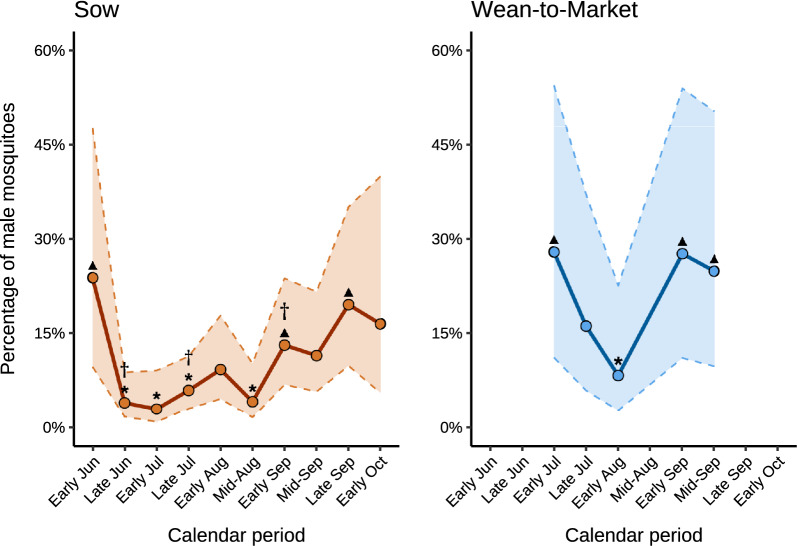
Model-adjusted mean percentage of male mosquitoes by calendar period on sow and wean-to-market farms. Circles (o) indicate the model-adjusted mean percentage of male mosquitoes ((number of males / total number aspirated [male + female] mosquitoes) × 100) collected with aspirators across habitat (water body and vegetation) and individual farms during each calendar period. Dashed lines (–) represent the 95% confidence intervals. Triangles (▲) indicate periods with significantly higher estimates than those marked with asterisks (*), while daggers (†) denote periods involved in multiple, related pairwise comparisons. Significant contrasts for sow farms were early June versus late June, early and late July, and mid-August (*P* < 0.05); early September versus late June and late July (*P* ≤ 0.03); late September versus late June, early and late July, and mid-August (*P* ≤ 0.03). Significant contrasts for wean-to-market farms were early August versus early July and early and mid-September (*P* ≤ 0.04). Data from the early and late June, mid-August, late September, and early October calendar periods for wean-to-market farms were excluded from the analysis owing to estimation issues caused by the absence of male mosquitoes near water bodies

The mean and range percentages of male mosquitoes collected using aspirators near water bodies and vegetation, for each calendar period across farms within each production system, are presented in Supplementary Tables 7 and 8. The two-way interaction between habitat and calendar period on male mosquito proportions was not statistically significant for sow (*P* = 0.15) and WTM (*P* = 0.14) farms. After dropping the interaction and evaluating the main effects, for sow farms, the model-adjusted percentage of male mosquitoes on sow farms was significantly higher in vegetation (20.1%; 95% CI 8.8–39.4%) than near water bodies (3.8%; 95% CI 1.5–9.8%) (*P* = 0.02). Additionally, the percentage of male mosquitoes also varied significantly by calendar period (*P* < 0.01; Fig. 2). Specifically, the percentage of male mosquitoes was significantly higher in early June and late September compared with late June, all of July, and mid-August (*P* < 0.05), and in early September compared with late June and late July (*P* ≤ 0.03). On WTM farms, the percentage of male mosquitoes was 31.0 (95% CI 8.0–69.8) in vegetation and 11.7 (95% CI 2.4–41.7) near water bodies (*P* = 0.21). However, the percentage of male mosquitoes differed significantly across calendar periods (*P* < 0.01; Fig. 2), with a lower percentage of male mosquitoes detected in early August compared with early July, and early and mid-September (*P* ≤ 0.04).

### Abundance, species diversity, and feeding patterns of blood-fed female mosquitoes

A total of 444 blood-fed female mosquitoes were captured with BG traps and/or aspirators across sow and WTM farms. Of these, 25.7% (114/444) were captured on sow farms, and 74.3% (330/444) were captured on WTM farms. On sow farms, 31.6% (36/114) of the blood-fed females were captured indoors, all of which fed on pigs (*Sus scrofa*). The remaining 68.4% (78/114) were captured outdoors, with 30.8% (24/78) captured near water bodies and 69.2% (54/78) in vegetation. Among those captured outdoors, 60.3% (47/78) fed on pigs, while 39.7% (31/78) fed on other hosts, including white-tailed deer (*Odocoileus virginianus,* *n* = 27), turkey vultures (*Cathartes aura*, *n* = 3), and cattle (*Bos taurus,* *n* = 1). On WTM farms, 91.8% (303/330) of the blood-fed females were captured indoors, where 95% (288/303) fed on pigs, while 5% (15/303) fed on other hosts, including white-tailed deer (*n* = 6), cattle (*n* = 5), turkey vulture (*n* = 2), and American robins (*n* = 2). Overall, 27 (27/330) of the blood-fed females (8.2%) were captured outdoors, with 29.6% (8/27) captured near water bodies and 70.4% (19/27) in vegetation. Of the total blood-fed females captured outdoors, 51.9% (14/27) fed on pigs and 48.1% (13/27) fed on other hosts, including white-tailed deer (*n* = 5), cattle (*n* = 5), turkey vultures (*n *= 2), and Swainson’s thrush (*Catharus ustulatus*, *n* = 1).

Overall, 7 unique blood-fed mosquito species were identified on sow farms and 12 on WTM farms. The dominant species on sow farms were *Aedes vexans* (*n* = 50), *Culex salinarius* (*n* = 32), *Culex pipiens* (*n* = 16), and *Anopheles quadrimaculatus* (*n* = 9), whereas on WTM farms, the most abundant species were *Anopheles quadrimaculatus* (*n* = 148), *Culex pipiens* (*n* = 88), and *Anopheles punctipennis* (*n* = 44).

### Blood-fed female mosquito abundance by location and calendar period on sow and WTM farms

**Fig. 3:**
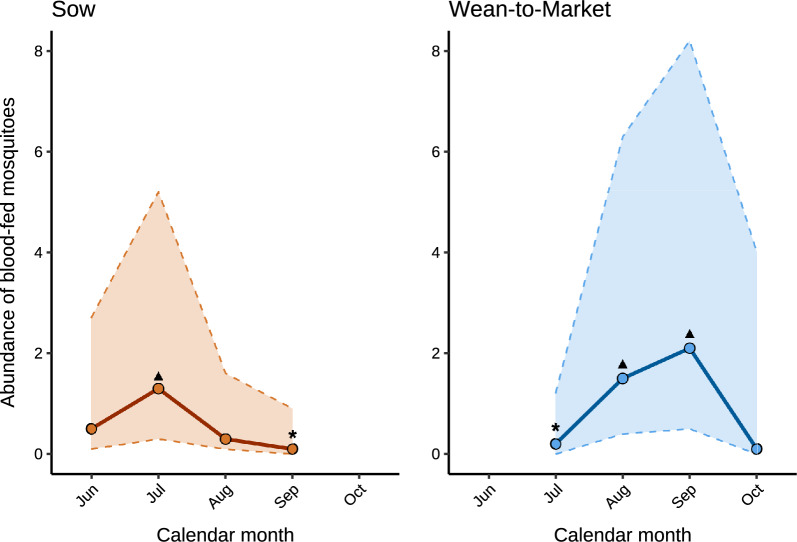
Model-adjusted mean counts of blood-fed female mosquitoes by calendar month for sow and wean-to-market farms. Circles (o) indicate mean blood-fed female mosquito counts trapped with BG traps across locations (indoors and outdoors) and individual farms during each calendar month. Dashed lines (–) represent the 95% confidence intervals. The significant contrast for sow farms was July versus September (*P* = 0.02). Triangles (▲) indicate periods with significantly higher estimates than those marked with asterisks (*). Significant contrasts for wean-to-market farms were July versus August and September (*P* ≤ 0.03). Owing to estimation issues, calendar months with no blood-fed mosquito captures, such as October for sow farms and June for wean-to-market farms, were excluded from the analysis

The proportion and percentage of blood-fed female mosquitoes captured using BG traps and/or aspirators indoors, near water bodies, and in vegetation for each calendar period across farms is provided in Supplementary Tables 9 and 10. On sow farms, the abundance of blood-fed female mosquitoes did not differ significantly by location (*P* = 0.20; Table 1); however, it did differ significantly by calendar month (*P* = 0.02), where abundance was higher in July compared with September (Fig. 3). On WTM farms, the abundance differed significantly by location, where it was higher indoors compared with outdoors (*P* < 0.01; Table 1). Similarly, the abundance of blood-fed mosquitoes differed significantly by calendar month, where it was higher in August and September compared with July (*P* < 0.01; Fig. 3).

**Table 1:** Model-adjusted mean count of blood-fed female mosquitoes by location across sow and wean-to-market farms

Variable	Model-adjusted mean blood-fed female counts^1^	95% CI	*P*-value
Sow			0.20
Indoor	0.5 ^a^	0.1–1.9	
Outdoor	0.3 ^a^	0.1–1.2	
Wean-to-market			< 0.01
Indoor	1.8 ^a^	0.3–9.6	
Outdoor	0.1 ^b^	0.0–0.7	

Different superscript letters indicate significant pairwise differences at *P* < 0.05

^1^Mean counts refer to mean blood-fed female mosquito counts from specimens trapped with BG traps indoors and outdoors across farms within each production system (sow and WTM farms)

*CI* confidence interval

### Probability of mosquitoes feeding on pig versus other hosts by location and calendar period on sow and WTM farms

**Fig. 4:**
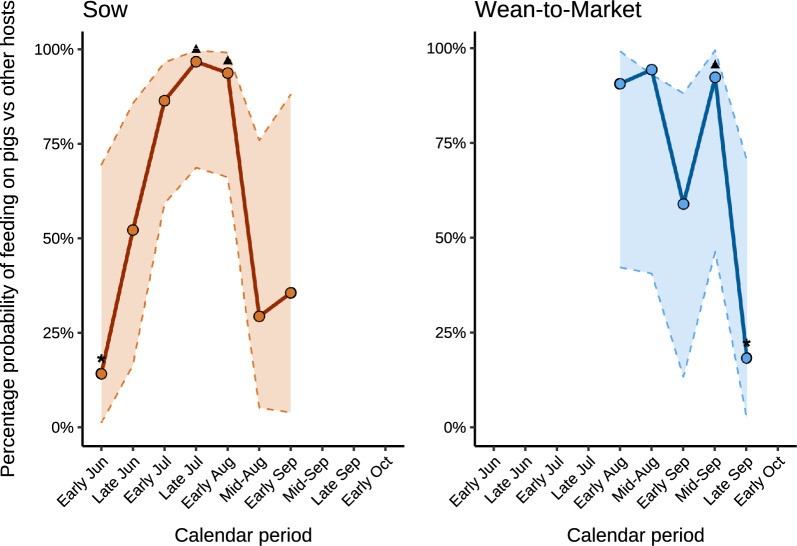
Model-adjusted percent probability of mosquitoes feeding on swine versus other hosts by calendar period on sow and wean-to-market farms. Circles (o) indicate the model-adjusted mean percent probability of mosquitoes feeding on pigs versus other hosts across location (indoors and outdoors [water body and vegetation]) and farms during each calendar period. Dashed lines (–) represent the 95% confidence intervals. Triangles (▲) indicate periods with significantly higher estimates than those marked with asterisks (*). Significant contrasts for sow farms were early June versus late July and early August (*P* ≤ 0.04). The significant contrast for wean-to-market farms was mid-September versus late September (*P* < 0.03). Owing to estimation issues, calendar periods with absent or very few blood-fed mosquito captures in mid and late September and early October for sow farms, and early/late June, early/late July, and early October for wean-to-market farms, were excluded from the analysis

The proportion of blood-fed female mosquitoes captured with BG traps and/or aspirators indoors, near water bodies, and in vegetation for each host species across farms is provided in Supplementary Tables 11 and 12. On sow farms, the probability of mosquitoes feeding on pigs versus other hosts did not differ significantly by mosquito species (*P* = 0.15; Table 2), but did differ by calendar period (*P* < 0.05; Fig. 4), with higher probabilities observed in early and late July, and early August compared with early June. Similarly, on WTM farms, the probability of mosquitoes feeding on pigs versus other hosts did not vary significantly by mosquito species (*P* = 0.67; Table 2) but did differ by calendar period (*P* ≤ 0.01; Fig. 4) and location (*P* ≤ 0.01; Table 2). The probability of mosquitoes feeding on pigs versus other hosts was higher in early and mid-September compared with late September. The probability was also higher indoors compared with outdoors.

**Table 2:** Model-adjusted mean percent probability of mosquitoes feeding on swine versus other hosts by mosquito species and location across sow and wean-to-market farms

Variable	Mean percent probability (%) ^1^	95% CI (%)	*P*-value
Sow			
Mosquito species			0.15
*Aedes vexans*	34.8 ^a^	11.8–67.9	
*Anopheles quadrimaculatus*	65.6 ^a^	21.3–93.1	
*Culex pipiens*	45.4 ^a^	13.0–82.2	
*Culex salinarius*	75.4 ^a^	36.2–94.3	
Others	92.0 ^a^	35.7–99.5	
Wean-to-market			
Mosquito species			0.67
*Anopheles punctipennis*	73.8 ^a^	18.0–97.3	
*Anopheles quadrimaculatus*	78.0 ^a^	27.1–97.1	
*Culex pipiens*	88.1 ^a^	44.2–98.6	
Others	69.8 ^a^	21.2–95.2	
Location			
Indoors	97.4 ^a^	80.9–99.7	< 0.01
Outdoors	24.1 ^b^	2.8–80.6	

Different superscript letters indicate significant pairwise differences at *P* < 0.05

^1^Mean probability refers to the mean probability (in %) of mosquitoes feeding on pigs versus other hosts by mosquito species and location across farms within each production system (sow and WTM farms). Estimates were derived from two separate models—one fitted for sow farms and another for WTM farms—since production system was not included as an explanatory variable

*CI* confidence interval

## Discussion

To the best of our knowledge, this study offers the first comprehensive baseline report of mosquito habitat preferences and feeding patterns on commercial swine farms in the USA. Our findings revealed: (1) The abundance of resting mosquitoes varied significantly over time on both sow and WTM farms; (2) vegetation surrounding swine facilities harbored significantly more resting mosquitoes on WTM farms, and a higher proportion of male mosquitoes on sow farms; (3) while no difference was observed on sow farms, the abundance of blood-fed female mosquitoes and the probability of mosquitoes feeding on pigs versus other host species was significantly higher indoors compared with outdoors on WTM farms; (4) the abundance of blood-fed female mosquitoes and the probability of mosquitoes feeding on pigs versus other hosts varied significantly over time on both sow and WTM farms; and (5) a majority of the blood-fed female mosquitoes captured outdoors on both sow and WTM farms fed on pigs, whereas a subset of those trapped indoors on WTM farms fed on non-swine hosts.

Resting habitats serve as critical ecological niches where mosquitoes digest blood meals, complete gonotrophic cycles, and seek protection from environmental stressors following host feeding [26]. In this study, we assessed resting habitat preferences by quantifying the total number of resting mosquitoes (both males and females). Notably, this represents one of the first reports describing the distribution of male mosquitoes within a livestock production setting. The abundance of male mosquitoes was used as a proxy for identifying preferred mosquito habitats, given that males typically remain near larval habitats or the mammalian hosts, and exhibit limited dispersal compared with females [27]. While statistical significance varied among these indicators, a consistent pattern emerged: Vegetation supported higher numbers of resting and male mosquitoes compared with water body habitats on both sow and WTM farms. In addition, more blood-fed female mosquitoes were captured in vegetation compared with near water bodies on both sow and WTM farms. Because blood-fed females are typically found in locations used for post-feeding rest and digestion, their predominance in vegetation habitats provides further evidence that vegetation represents the primary resting sites for mosquitoes on commercial swine farms. This finding is also in agreement with prior studies reporting that vegetated areas provide favorable microclimatic conditions, such as shade, elevated humidity, and shelter [26, 28], that promote mosquito survival and resting behavior. These conditions may, in turn, enhance mosquito longevity and vectorial capacity, with important implications for arbovirus transmission dynamics in livestock production systems. In addition, the abundance of resting mosquitoes varied through time, peaking in late July on both sow and WTM farms. Seasonal fluctuations in mosquito populations are commonly associated with temperature and rainfall, which influence larval habitat availability, adult survival, and host-seeking behavior [29–31]. High summer temperatures can drive mosquitoes to shaded, humid resting sites to minimize physiological stress, while rainfall replenishes larval habitats and can synchronize emergences of new adults [13, 14, 32]. Collectively, these findings provide evidence of both where (vegetation habitats) and when (peak summer months) mosquito control efforts should be prioritized.

Another important finding from this study is the presence of blood-fed female mosquitoes inside barns on all the pig farms, regardless of production system, facility structure type, or biosecurity level. Unexpectedly, the abundance of blood-fed females was higher indoors compared with outdoors on WTM farms. Additionally, the probability of mosquitoes feeding on pigs versus other hosts was higher indoors compared with outdoors. These findings were not surprising owing to the abundance of host species (pigs) inside the barns and the use of natural (curtain) ventilation, which supports easy mosquito movements into and out of the WTM pig barns. In contrast, no location-based differences in the abundance of blood-fed mosquitoes and the probability of mosquitoes feeding on pigs versus other hosts were observed on sow farms, likely because the solid-wall construction and the negative-pressure mechanical ventilation system of the sow barns present a more substantial physical barrier to mosquito movement than the WTM barns. While these physical barriers mentioned above do not entirely exclude insects from the barns, as insects were occasionally observed inside sow barns, the tendency of mosquitoes to select pigs over other hosts indicates that mosquito movement into or out of the barns is likely minimal. Differences in sampling approaches may also have influenced observed patterns. CO_2_-baited traps were used outdoors to enhance capture of host-seeking mosquitoes in open environments where host cues are more diffuse, whereas CO_2_ was excluded indoors because the pigs in the swine barns likely generate elevated ambient CO_2_ levels that could reduce the effectiveness of supplemental dry ice. Likewise, UV light was not used outdoors because its attractiveness may be diminished under natural lighting conditions, particularly during clear evenings or periods of moonlight, while CO_2_ remains an effective attractant for host-seeking mosquitoes. Furthermore, the abundance of blood-fed females also varied through time, peaking in July on sow farms and in September on WTM farms. Although these differences were statistically significant, the biological relevance of a change from roughly one to two blood-fed mosquitoes per month should be interpreted cautiously. Moreover, these differences may reflect variation in host availability, particularly on WTM farms, where pigs are marketed at different times in a production cycle depending on live weight. In June through to mid-August, marketing events were monitored during site visits across all WTM farms. Concurrently, the barns underwent pressure washing and disinfection as part of the preparatory measures for the next production cycle. Such turnover alters host density and accessibility, thereby influencing mosquito feeding activity. The peak in mosquito feeding observed on WTM farms in September may partly reflect farm-level differences in production and marketing schedules. For instance, one WTM farm marketed its pigs early in the sampling period (June), while three others marketed later, around early and mid-August. As a result, barns that were repopulated with a new cohort of pigs later in the summer may have provided renewed host availability and suitable mosquito environment, contributing to the September population peak. Conversely, this pattern is not frequently observed on sow farms as each farm is consistently operational except for unforeseen circumstances such as disease outbreaks. Furthermore, these seasonal peaks coincided with periods when the probability of mosquitoes feeding on pigs versus other hosts was significantly higher in late July and early August on sow farms, and mid-September on WTM farms, further supporting our hypothesis that production cycles and the timing of pig sales are key drivers of these observed patterns. Collectively, these findings are of particular concern because summer and early fall correspond to periods of elevated arboviral transmission risk in livestock environments [33]. As such, continued mosquito access to pigs and potential pathogen transmission could be detrimental to swine producers and the swine industry at large.

It was not surprising that most of the mosquitoes captured indoors on both sow and WTM farms fed on pigs. However, our findings also revealed that the majority of blood-fed mosquitoes captured outdoors on these farms fed on pigs, whereas a subset of those trapped indoors on WTM farms had blood meals from non-swine hosts (white-tailed deer, cattle, turkey vultures, and American robins). As noted earlier, this may be owing to the structural design of sow and WTM facilities, where curtain-sided barns and natural ventilation systems of WTM farms likely do not impair the entry and exit of mosquitoes. Another important consideration is the possibility of sequential feeding: Mosquitoes may begin feeding on non-swine hosts outdoors and then move indoors to feed on pigs, or vice versa. Because Sanger sequencing detects only the dominant host DNA [34], such mixed or incomplete blood meals are difficult to capture, which may explain why some mosquitoes collected indoors had non-swine blood meals. In contrast, sow barns are more enclosed and operate under negative-pressure ventilation, which likely restricts the entry of outside mosquitoes and helps explain why all mosquitoes trapped indoors had fed exclusively on pigs. This restricted entry alone, however, does not fully account for the high proportion of pig-derived blood meals observed outdoors. The detection of male mosquitoes inside barns (data not shown) further raises the possibility of cryptic larval habitats within buildings or beneath manure pits. If mosquitoes are emerging from these sites, newly eclosed females would be expected to take their first blood meal on pigs indoors and then exit through ventilation openings, providing a biologically plausible explanation for the abundance of pig-derived blood meals in mosquitoes captured outdoors. Ultimately, these findings imply that while structural biosecurity breaches may be more evident in WTM farms, sow farms cannot be overlooked [35]. Sow farms typically house breeding stock, gestating sows, and neonatal piglets, which represent immunologically vulnerable populations more prone to severe disease outcomes [36, 37]. Furthermore, the interconnected and confined structures of sow facilities may facilitate rapid within-farm pathogen spread if biosecurity measures fail. Thus, even though WTM farms may appear structurally more permeable to mosquito entry, biosecurity vigilance remains equally—if not more—critical in sow operations to mitigate the potential for pathogen introduction and transmission [38].

Although the probability of feeding on pigs versus other hosts did not differ significantly across mosquito species, several competent arbovirus vectors, including dominant species such as *Culex pipiens*, *Culex salinarius*, *Anopheles quadrimaculatus*, *Anopheles punctipennis*, and *Aedes vexans* [39], fed opportunistically on both domestic livestock and wildlife. Notably, among the non-swine blood meals identified indoors on WTM farms was the American robin, a recognized amplifying host for WNV and SLEV that plays a key role in maintaining these viruses in the environment [40]. Although infections with these arboviruses are generally subclinical in pigs [41], *Culex* mosquitoes can become infective after feeding on robins [40], enabling transmission to humans and other vertebrate hosts [42]. We also detected blood meals from Swainson’s thrush in *Culex* mosquitoes collected outdoors on WTM farms. While pathogen testing was not performed in this study, Swainson’s thrush has previously been reported as susceptible to WNV [43], and its long-distance migratory behavior suggests potential for geographic dispersal of arboviruses, even though its role as an amplifying host remains unclear. Furthermore, Turkey vulture (*Cathartes aura*) blood meals were detected in both *Culex* and *Anopheles* mosquitoes. Although this species is susceptible to WNV [44], there is no evidence that it plays a major role in virus transmission. Its presence nonetheless underscores the diversity of avian hosts interacting with mosquitoes around swine farms. Other vertebrate hosts identified, including cattle and white-tailed deer, are likewise susceptible to WNV infection but are generally considered dead-end hosts that contribute minimally to virus maintenance or transmission [45]. Collectively, these findings highlight the need for a holistic approach to mosquito control in pig production systems, one that considers not only pig–mosquito interactions but also the presence and management of other vertebrate hosts near swine facilities.

Some limitations of this study include: (1) BG-Pro traps may exhibit taxon-specific sampling bias, as trap performance can vary among mosquito species and physiological states, potentially influencing observed patterns of abundance and feeding behavior, and (2) the lack of pathogen screening limited our ability to directly assess associations between blood-fed mosquitoes and pathogen transmission risk. Detecting arboviruses in mosquitoes would require substantially larger sample sizes, as these pathogens typically circulate at very low prevalence in swine populations and, consequently, in mosquito vectors; (3) this study was geographically restricted to commercial swine farms in Southeast Iowa, which may limit the generalizability of results to other regions with different climatic conditions, farm management practices, or mosquito communities. Additionally, mosquito feeding patterns were not assessed across the entire year, as sampling within barns was not conducted in June owing to biosecurity constraints, and field collections ended in mid-October, and (4) microclimatic variables (e.g., temperature, humidity, and precipitation) were not incorporated into the analyses and may have influenced mosquito activity and habitat use.

In addition to the need for future research to provide a more comprehensive assessment of mosquito–host interactions in and around pig farms, future studies examining whether mosquito vectors, which primarily feed on domestic livestock such as pigs, maintain their preference for a pig blood meal to facilitate their reproductive cycle even after initially feeding on a different host are needed. The current study indicates that a considerable proportion of host-seeking mosquitoes that had a non-swine blood meal were captured indoors, implying that specific mosquito species persist in seeking out swine hosts despite the opportunity to feed on other animals. This aspect warrants additional attention as it could elucidate discrepancies in blood-feeding behaviors among various species, particularly in the context of sow and WTM farms.

## Conclusions

This study provides the first comprehensive evidence on the habitat preferences and feeding behavior of mosquito vectors on commercial swine farms in the USA. While the abundance of resting mosquitoes varied over time, vegetation habitats supported significantly higher numbers of resting mosquitoes on WTM farms and a higher proportion of male mosquitoes on sow farms, indicating that vegetation may serve as key resting sites for local mosquito populations. Furthermore, the detection of pig blood meals in mosquito species known to be arbovirus vectors, captured both indoors and outdoors during high-risk months, highlights an opportunity to improve insect biosecurity on swine farms. The concurrent feeding on non-swine hosts, including wildlife, underscores the potential risk mosquitoes pose for disease introduction and spread. Routine mosquito vector surveillance and targeted control measures, both indoors and in surrounding habitats, should be integrated into herd biosecurity programs to reduce mosquito-related health risks in commercial swine operations.

## Supplementary Information

Supplementary Material 1. **Table S1**. Explanations of outcomes and explanatory variables used in the study. **Table S2**. Distance between swine farms within each production system. **Table S3**. Distance between trap locations for each farm on sow and wean-to-market farms. **Table S4**. PCR types and their primer combinations, sequence information, and thermal profiles. **Table S5**. The total, mean, and range of the number of mosquitoes (including both males and females) captured near water bodies and in vegetation by aspiration by calendar period across farms within production system for sow farms. **Table S6**. The total, mean, and range of the number of mosquitoes (including both males and females) captured near water bodies and vegetation by aspiration by calendar period across farms within production system for wean-to-market farms. **Table S7**. The proportion (in %) of male mosquitoes captured by aspiration by calendar period and habitat (water body versus vegetation) across farms within production system for sow farms. **Table S8**. The proportion (in %) of male mosquitoes captured by aspiration by calendar period and habitat (water body versus vegetation) across farms within production system for wean-to-market farms. **Table S9**. The proportion and percentage of blood-fed mosquitoes captured by BG traps and/or aspirators by calendar period and location (indoor/water body/vegetation) across sow farms. **Table S10**. The total proportion and percentage of blood-fed mosquitoes captured by BG traps and/or aspirators by calendar period and location (indoor/water body/vegetation) across wean-to-market farms. **Table S11**. Total number of host species identified and the proportion of blood-fed mosquitoes feeding on each host species, stratified by location (indoors, near water bodies, and vegetation) and capture method (BG traps and/or aspirators) on sow farms. **Table S12**. Total number of host species identified and the proportion of blood-fed mosquitoes feeding on each host species, stratified by location (indoors, near water bodies, and vegetation) and capture method (BG traps and/or aspirators) on wean-to-market farms.

## Data Availability

The data supporting the conclusions of this article are included within the article and its additional files.

## Abbreviations

JEV: Japanese encephalitis virus
SLEV: Saint Louis encephalitis virus
WNV: West Nile virus
RVFV: Rift Valley fever virus
PCR: Polymerase chain reaction
FTA: Fast technology of analysis of nucleic acid
WTM: Wean-to-market
BG: BG-Pro light traps (Biogents AG, Regensberg, Germany)
*COI*: Cytochrome c oxidase subunit I gene
CI: Confidence interval
NCBI: National Center for Biotechnology Information
BLAST: Basic Local Alignment Search Tool
DNA: Deoxyribonucleic acid
