# Supplementary material for: Investigating habitat preferences and host-feeding behavior of mosquito disease vectors on commercial swine farms in the USA
Source: Parasit Vectors. 2026 Mar 12;19:173. doi: 10.1186/s13071-026-07349-6 (PMC13097815; doi:10.1186/s13071-026-07349-6)
Supplement: Supplementary file 1 — Supplementary Material 1. Table S1. Explanations of outcomes and explanatory variables used in the study. Table S2. Distance between swine farms within each production system. Table S3. Distance between trap locations for each farm on sow and wean-to-market farms. Table S4. PCR types and their primer combinations, sequence information, and thermal profiles. Table S5. The total, mean, and range of the number of mosquitoes (including both males and females) captured near water bodies and in vegetation by aspiration by calendar period across farms within production system for sow farms. Table S6. The total, mean, and range of the number of mosquitoes (including both males and females) captured near water bodies and vegetation by aspiration by calendar period across farms within production system for wean-to-market farms. Table S7. The proportion (in %) of male mosquitoes captured by aspiration by calendar period and habitat (water body versus vegetation) across farms within production system for sow farms. Table S8. The proportion (in %) of male mosquitoes captured by aspiration by calendar period and habitat (water body versus vegetation) across farms within production system for wean-to-market farms. Table S9. The proportion and percentage of blood-fed mosquitoes captured by BG traps and/or aspirators by calendar period and location (indoor/water body/vegetation) across sow farms. Table S10. The total proportion and percentage of blood-fed mosquitoes captured by BG traps and/or aspirators by calendar period and location (indoor/water body/vegetation) across wean-to-market farms. Table S11. Total number of host species identified and the proportion of blood-fed mosquitoes feeding on each host species, stratified by location (indoors, near water bodies, and vegetation) and capture method (BG traps and/or aspirators) on sow farms. Table S12. Total number of host species identified and the proportion of blood-fed mosquitoes feeding on each host species, stratified by loc [file 13071_2026_7349_MOESM1_ESM.docx]

**Supplementary materials**

**Supplementary Table 1**. Descriptions of outcomes and explanatory variables used in the study.

| Variables | Description |
| --- | --- |
| Outcomes |  |
| Resting mosquito abundance | The total number of mosquitoes (males and females) captured using aspirators in vegetation and water body habitats, representing resting populations. This is presented as mean mosquito counts across farms within each production system. |
| Proportion of male mosquitoes | The proportion (measured in %) of male mosquitoes, calculated as (number of males ÷ total mosquitoes aspirated [males + females]) × 100. |
| Abundance of blood-fed female mosquitoes | The total number of mosquitoes (females) that have taken a bloodmeal from a host species, captured using BG traps and/or aspirators indoors and outdoors (near water bodies and in vegetation). This is presented as the mean number of blood-fed females across farms within each production system. |
| Probability of feeding on pigs vs other hosts | This refers to the probability (likelihood) of mosquitoes–captured using traps and/or aspirators–feeding on pigs compared to other hosts. This is presented as the mean probability of mosquitoes feeding on pigs vs other hosts, across farms within each production system. |
| Explanatory variables |  |
| Calendar period | This refers to the sampling intervals:   - early June (June 6–7, 2024), - late June (June 20–21, 2024), - early July (July 4–5, 2024), - late July (July 18–19, 2024), - early August (August 1–2, 2024), - mid-August (August 15–16, 2024), - early September (September 5–6, 2024), - mid-September (September 12–13, 2024), - late September (September 26–27, 2024) - early October (October 10–11, 2024). |
| Calendar month | This refers to sampling intervals aggregated into months to address the abundance of blood-fed female mosquitoes captured over time:   - June (6 – 7 **\|** 20 – 21, 2024) - July (4 – 5 **\|** 18 – 19, 2024) - August (1 – 2 **\|** 15 – 16, 2024) - September (5 – 6 **\|** 12 – 13 **\|** 26 – 27, 2024) - October (10 – 11, 2024) |
| Mosquito species | This refers to the unique mosquito species identified to have taken a blood meal. Because some mosquito species had few observations, they were grouped as *Culex pipiens, Culex salinarius, Aedes vexans, Anopheles quadrimaculatus,* and others (*Culex erraticus, Culex tarsalis,* and *Anopheles punctipennis*) for sow farms, and *Anopheles quadrimaculatus,* *Culex pipiens, Anopheles punctipennis,* and others (*Psorophora columbiae, Aedes dorsalis, Aedes japonicus, Aedes vaxans, Culex erraticus, Culex restuans, Culex salinarius, Culex tarsalis,* and *Ochlerotatus trivittatus*). |
| Location | This refers to the general placement of mosquito traps as either indoors (e.g., inside gestation barns of sow farms) or outdoors (near water bodies and in vegetation), where both traps and aspirators were used. |
| Habitat | This refers to the specific outdoor environment where traps were set and aspirators used to capture mosquitoes, categorized as either near water bodies (lagoons and ponds) or vegetation areas. |

**Supplementary Table 2.** Distance between swine farms within each production system.

| Production system | Farms | Distance (km) | Average distance (km) |
| --- | --- | --- | --- |
| Sow | SF1 – SF2 | 6.1 | 7.6 |
|  | SF1 – SF3 | 4.8 |  |
|  | SF1 – SF4 | 2.1 |  |
|  | SF1 – SF5 | 13.8 |  |
|  | SF2 – SF3 | 5.3 |  |
|  | SF2 – SF4 | 5.8 |  |
|  | SF2 – SF5 | 7.7 |  |
|  | SF3 – SF4 | 4.5 |  |
|  | SF3 – SF5 | 12.9 |  |
|  | SF4 – SF5 | 13.4 |  |
| Wean-to-market | WF1 – WF2 | 1.6 | 20.8 |
|  | WF1 – WF3 | 3.4 |  |
|  | WF1 – WF4 | 27.4 |  |
|  | WF1 – WF5 | 30.6 |  |
|  | WF2 – WF3 | 4.7 |  |
|  | WF2 – WF4 | 28.0 |  |
|  | WF2 – WF5 | 29.6 |  |
|  | WF3 – WF4 | 31.4 |  |
|  | WF3 – WF5 | 33.8 |  |
|  | WF4 – WF5 | 17.7 |  |

SF = Sow farm; WF = Wean-to-market farm

Distance (km) was defined as the straight-line distance between swine farms within the same production system, calculated in Google Earth using the geographic coordinates of each farm.

Average distance (km) represents the mean of all unique pairwise inter-farm distances within each production system (i.e., sow farms or wean-to-market farms).

**Supplementary Table 3.** Distance between trap locations for each farm on **sow** and **wean-to-market** farms.

| Production system | Farms | Trap locations | Distance (meters) |
| --- | --- | --- | --- |
| Sow | SF1 | Indoor to Water body | 149.8 |
|  |  | Indoor to Vegetation | 350.8 |
|  |  | Water body to Vegetation | 274.6 |
|  | SF2 | Indoor to Water body | 138.9 |
|  |  | Indoor to Vegetation | 329.6 |
|  |  | Water body to Vegetation | 157.3 |
|  | SF3 | Indoor to Water body | 144.8 |
|  |  | Indoor to Vegetation | 233.0 |
|  |  | Water body to Vegetation | 100.5 |
|  | SF4 | Indoor to Water body | 146.8 |
|  |  | Indoor to Vegetation | 209.8 |
|  |  | Water body to Vegetation | 86.4 |
|  | SF5 | Indoor to Water body | 115.0 |
|  |  | Indoor to Vegetation | 295.1 |
|  |  | Water body to Vegetation | 398.9 |
| Wean-to-market | WF1 | Indoor to Vegetation | 34.4 |
|  | WF2 | Indoor to Vegetation | 57.4 |
|  | WF3 | Indoor to Water body | 109.2 |
|  |  | Indoor to Vegetation | 308.2 |
|  |  | Water body to Vegetation | 203.8 |
|  | WF4 | Indoor to Water body | 293.2 |
|  |  | Indoor to Vegetation | 502.1 |
|  |  | Water body to Vegetation | 461.0 |
|  | WF5 | Indoor to Water body | 99.4 |
|  |  | Indoor to Vegetation | 365.2 |
|  |  | Water body to Vegetation | 272.3 |

SF = Sow farm; WF = Wean-to-market farm.

SF1, SF2, WF3, WF4, and WF5 consist of ponds as water body habitats, while SF3, SF4, and SF5 consist of sewage lagoons as water body habitats. WF1 and WF2 did not have water body habitats.

Vegetation habitats consisted of trees, dense shrubs, and tall grass on SF1–SF5 and WF2–WF5, whereas vegetation habitat on WF1 consisted primarily of corn fields.

Distance (m) was defined as the straight-line distance between mosquito trap locations within the same farm, calculated in Google Earth using the geographic coordinates recorded at the time of trap placement. Distances were computed for all relevant within-farm pairs, including indoor traps and traps placed near vegetation or water bodies, as well as between vegetation and water-body trap locations when both were present.

Across all farms, the average distance between indoor traps and traps placed near water bodies was 149.6 m, between indoor and vegetation traps was 268.7 m, and between vegetation and water-body traps was 244.4 m.

**Supplementary Table 4**. PCR types and their primer combinations, sequence information, and thermal profiles.

| PCR type | Primer combination | Primer sequence | Thermal profiles |
| --- | --- | --- | --- |
| Vertebrates (host) *^1^* |  |  |  |
| PCR A | VertCOI_7194_F | 5′-CGMATRAAYAAYATRAGCTTCTGA Y-3′ | 1 cycle of 95°C for 3 min; 5 cycles of 95°C for 40 s, 45°C for 30 s, 72°C for 3 min; then 35 cycles of 95°C for 40 s, 49°C for 30 s, 72°C for 3 min; finally, 1 cycle of 72°C for 7 min. |
|  | Mod_RepCOI_R | 5′-TTCDGGRTGNCCRAARAATCA-3′ |  |
| PCR B | Mod_RepCOI_F | 5′-TNTTYTCMACYAACCACA AAG A-3′ | 1 cycle of 95°C for 3 min; 5 cycles of 95°C for 40 s, 45°C for 30 s, 72°C for 3 min; then 35 cycles of 95°C for 40 s, 46°C for 30 s, 72°C for 3 min; finally, 1 cycle of 72°C for 7 min. |
|  | VertCOI_7216_R | 5′-CARAAGCTYATGTTRTTYATDCG-3′ |  |
| PCR C | VertCOI_10096_F | 5′-CHCAATACCAAACNCCHYTNTTYG-3′ | 1 cycle of 95°C for 3 min; 40 cycles of 95°C for 40 s, 48°C for 30 s, 72°C for 3 min; finally, 1 cycle of 72°C for 7 min. |
|  | Mod_RepCOI_R | 5′-TTCDGGRTGNCCRAARAATCA-3′ |  |
| Invertebrates (Mosquito) *^2^* | LCO1490 | 5′-GGTCAACAAATCATAAAGATATTGG-3′ | 1 cycle of 94°C for 1 min; 5 cycles of 94°C for 30 s, 45°C for 40 s, and 72°C for 1 min; followed by 35 cycles of 94°C for 30 s, 51°C for 40 s, and 72°C for 1 min; finally, 1 cycle of 72°C for 10 min. |
|  | HCO2198 | 5′-TAAACTTCAGGGTGACCAAAAAATCA-3′ |  |

*^1^* Each 20 µL reaction consisted of 10 µL of 2× Apex Taq RED Master Mix, 0.375 µM of each primer, 1 µL of DNA template, and ultrapure water to a final volume of 20 µL.

*^2^* Each 20 µL reaction contained 10 µL of 2× Apex Taq RED Master Mix, 1 µM of each primer, 1 µL DNA template, and 1 µL of DNA template, and ultrapure water to a final volume of 20 µL.

**Supplementary Table 5.** The total, mean, and range of the number of mosquitoes (including both males and females) captured near water bodies and in vegetation by aspiration by calendar period across farms within production system for **sow farms**.

| Calendar period | Water bodies | | | | | Vegetation | | | | |
| --- | --- | --- | --- | --- | --- | --- | --- | --- | --- | --- |
|  | Farm; N *^1^* | Aspirator; N *^2^* | Total *^3^* | Mean *^3^* | Range *^4^* | Farm; N *^1^* | Aspirator; N *^2^* | Total *^3^* | Mean *^3^* | Range *^4^* |
| Early June (June 6 – 7) | 4 | 8 | 8 | 1.1 | 0 – 7 | 4 | 8 | 36 | 6.9 | 0 – 26 |
| Late June (June 20 – 21) | 4 | 8 | 62 | 8.1 | 0 – 44 | 4 | 8 | 101 | 15.5 | 1 – 51 |
| Early July (July 4 – 5) | 4 | 8 | 25 | 5.4 | 0 – 15 | 4 | 8 | 73 | 14.1 | 1 – 37 |
| Late July (July 18 – 19) | 4 | 8 | 176 | 28.2 | 0 – 168 | 4 | 8 | 447 | 96.5 | 22 – 171 |
| Early August (August 1 – 2) | 4 | 8 | 129 | 31.8 | 0 – 117 | 4 | 8 | 147 | 27.0 | 10 – 51 |
| Mid-August (August 15 – 16) | 4 | 8 | 154 | 25.9 | 0 – 127 | 4 | 8 | 46 | 11.0 | 0 – 39 |
| Early September (September 5 – 6) | 4 | 8 | 137 | 32.0 | 5 – 75 | 4 | 8 | 112 | 26.9 | 2 – 53 |
| Mid-September (September 12 – 13) | 4 | 8 | 105 | 26.1 | 1 – 83 | 4 | 8 | 115 | 26.0 | 1 – 55 |
| Late September (September 26 – 27) | 4 | 8 | 66 | 10.0 | 0 – 44 | 4 | 8 | 53 | 11.6 | 2 – 36 |
| Early October (October 10 – 11) | 4 | 8 | 10 | 2.0 | 0 – 6 | 4 | 8 | 9 | 1.5 | 0 – 8 |
| Total |  |  | 872 |  |  |  |  | 1,139 |  |  |

*^1^* Number of farms sampled during that calendar period.

*^2^* Number of aspirations across all sow farms during each calendar period per habitat.

*^3^* The total and mean number of mosquitoes captured across all aspirations/farms within each habitat.

*^4^* The range represents the minimum and maximum number of mosquitoes collected using aspirators during each calendar period within each habitat across all farms.

**Supplementary Table 6.** The total, mean, and range of the number of mosquitoes (including both males and females) captured near water bodies and vegetation by aspiration by calendar period across farms within production system for **wean-to-market farms**.

| Calendar period | Water bodies | | | | | Vegetation | | | | |
| --- | --- | --- | --- | --- | --- | --- | --- | --- | --- | --- |
|  | Farm; N *^1^* | Aspirator; N *^2^* | Total *^3^* | Mean *^3^* | Range *^4^* | Farm; N *^1^* | Aspirator; N *^2^* | Total *^3^* | Mean *^3^* | Range *^4^* |
| Early June (June 6 – 7) | 1 | 2 | 0 | 0.0 | 0 – 0 | 3 | 6 | 0 | 0.0 | 0 – 0 |
| Late June (June 20 – 21) | 1 | 2 | 0 | 0.0 | 0 – 0 | 3 | 6 | 14 | 2.3 | 0 – 14 |
| Early July (July 4 – 5) | 3 | 6 | 14 | 3.2 | 0 – 11 | 4 | 8 | 196 | 39.5 | 0 – 179 |
| Late July (July 18 – 19) | 3 | 6 | 24 | 5.2 | 0 – 14 | 4 | 8 | 203 | 45.2 | 0 – 94 |
| Early August (August 1 – 2) | 3 | 6 | 45 | 9.2 | 1 – 41 | 4 | 8 | 75 | 16.5 | 0 – 47 |
| Mid-August (August 15 – 16) | 3 | 6 | 2 | 0.7 | 0 – 2 | 4 | 8 | 96 | 14.9 | 0 – 65 |
| Early September (September 5 – 6) | 3 | 6 | 21 | 4.0 | 0 – 18 | 4 | 8 | 79 | 14.6 | 0 – 38 |
| Mid-September (September 12 – 13) | 3 | 6 | 31 | 5.3 | 0 – 24 | 4 | 8 | 164 | 28.0 | 0 – 72 |
| Late September (September 26 – 27) | 3 | 6 | 5 | 1.2 | 0 – 5 | 4 | 8 | 22 | 3.8 | 0 – 14 |
| Early October (October 10 – 11) | 3 | 6 | 1 | 0.3 | 0 – 1 | 4 | 8 | 6 | 0.8 | 0 – 4 |
| Total |  |  | 143 |  |  |  |  | 855 |  |  |

*^1^* Number of farms sampled during that calendar period.

*^2^* Number of aspirations across all wean-to-market farms during each calendar period per habitat.

*^3^* The total and mean number of mosquitoes captured across all aspirations/farms within each habitat.

*^4^* The range represents the minimum and maximum number of mosquitoes collected using aspirators during each calendar period within each habitat across all farms.

**Supplementary Table 7**. The proportion (in %) of male mosquitoes captured by aspiration by calendar period and habitat (water body vs. vegetation) across farms within production system for **sow farms**.

| Calendar period | Water bodies | | | | | Vegetation | | | | |
| --- | --- | --- | --- | --- | --- | --- | --- | --- | --- | --- |
|  | Farm; N *^1^* | Aspirator; N *^2^* |  | | | Farm; N *^1^* | Aspirator; N *^2^* |  | | |
|  |  |  | Mean % *^3^* |  | Range % *^4^* |  |  | Mean % *^3^* |  | Range % *^4^* |
| Early June (June 6 – 7) | 4 | 8 | 4.8 |  | 0.0 – 14.3 | 4 | 8 | 48.1 |  | 0.0 – 100.0 |
| Late June (June 20 – 21) | 4 | 8 | 1.4 |  | 0.0 – 10.0 | 4 | 8 | 8.6 |  | 0.0 – 100.0 |
| Early July (July 4 – 5) | 4 | 8 | 1.6 |  | 0.0 – 22.2 | 4 | 8 | 3.6 |  | 0.0 – 100.0 |
| Late July (July 18 – 19) | 4 | 8 | 6.1 |  | 0.0 – 66.7 | 4 | 8 | 9.4 |  | 0.0 – 100.0 |
| Early August (August 1 – 2) | 4 | 8 | 0.0 |  | 0.0 – 0.0 | 4 | 8 | 18.5 |  | 0.0 – 100.0 |
| Mid-August (August 15 – 16) | 4 | 8 | 0.3 |  | 0.0 – 4.8 | 4 | 8 | 4.6 |  | 0.0 – 100.0 |
| Early September (September 5 – 6) | 4 | 8 | 10.2 |  | 0.0 – 100.0 | 4 | 8 | 12.5 |  | 0.0 – 66.7 |
| Mid-September (September 12 – 13) | 4 | 8 | 9.8 |  | 0.0 – 100.0 | 4 | 8 | 22.7 |  | 0.0 – 100.0 |
| Late September (September 26 – 27) | 4 | 8 | 2.8 |  | 0.0 – 25.0 | 4 | 8 | 31.7 |  | 0.0 – 100.0 |
| Early October (October 10 – 11) | 4 | 8 | 3.3 |  | 0.0 – 16.7 | 4 | 8 | 35.7 |  | 0.0 – 100.0 |

*^1^* Number of farms sampled during that calendar period.

*^2^* Number of aspirations across all sow farms during each calendar period per habitat.

*^3^* The mean proportion (measured in %) of male mosquitoes, calculated as (number of males ÷ total mosquitoes aspirated [males + females]) × 100.

*^4^* The range represents the minimum and maximum number of male mosquitoes collected using aspirators during each calendar period within each habitat across all farms.

**Supplementary Table 8.** The proportion (in %) of male mosquitoes captured by aspiration by calendar period and habitat (water body vs. vegetation) across farms within production system for **wean-to-market farms**.

| Calendar period | Water bodies | | | | | Vegetation | | | | |
| --- | --- | --- | --- | --- | --- | --- | --- | --- | --- | --- |
|  | Farm; N *^1^* | Aspirator; N *^2^* | Mean % *^3^* |  | Range % *^4^* | Farm; N *^1^* | Aspirator; N *^2^* | Mean % *^3^* |  | Range % *^4^* |
| Late June (June 20 – 21) | 1 | 2 | 0.0 |  | 0.0 – 0.0 | 3 | 6 | 21.7 |  | 0.0 – 40.0 |
| Early July (July 4 – 5) | 1 | 6 | 5.3 |  | 0.0 – 33.3 | 4 | 8 | 23.7 |  | 0.0 – 100.0 |
| Late July (July 18 – 19) | 3 | 6 | 23.8 |  | 0.0 – 100.0 | 4 | 8 | 14.1 |  | 0.0 – 100.0 |
| Early August (August 1 – 2) | 3 | 6 | 2.6 |  | 0.0 – 33.3 | 4 | 8 | 11.3 |  | 0.0 – 100.0 |
| Mid-August (August 15 – 16) | 3 | 6 | 0.0 |  | 0.0 – 0.0 | 4 | 8 | 17.9 |  | 0.0 – 100.0 |
| Early September (September 5 – 6) | 3 | 6 | 1.0 |  | 0.0 – 11.1 | 4 | 8 | 19.9 |  | 0.0 – 100.0 |
| Mid-September (September 12 – 13) | 3 | 6 | 7.7 |  | 0.0 – 100.0 | 4 | 8 | 24.3 |  | 0.0 – 100.0 |
| Late September (September 26 – 27) | 3 | 6 | 0.0 |  | 0.0 – 0.0 | 4 | 8 | 24.4 |  | 0.0 – 100.0 |
| Early October (October 10 – 11) | 3 | 6 | 0.0 |  | 0.0 – 0.0 | 4 | 8 | 20.0 |  | 0.0 – 100.0 |

*^1^* Number of farms sampled during that calendar period.

*^2^* Number of aspirations across all wean-to-market farms during each calendar period per habitat.

*^3^* The mean proportion (measured in %) of male mosquitoes, calculated as (number of males ÷ total mosquitoes aspirated [males + females]) × 100.

*^4^* The range represents the minimum and maximum number of male mosquitoes collected using aspirators during each calendar period within each habitat across all farms.

**Supplementary Table 9**. The proportion and percentage of blood-fed mosquitoes captured by BG traps and aspirators by calendar period and location (indoor/water body/vegetation) across **sow farms**.

|  | Indoor | | | Water bodies | | | | | Vegetation | | | | |
| --- | --- | --- | --- | --- | --- | --- | --- | --- | --- | --- | --- | --- | --- |
| Calendar period | Farm; N *^1^* | Trap; N *^2^* | Proportion of blood-feds (%) *^3^* | Farm; N *^1^* | Trap; N *^2^* | Proportion of blood-feds (%) *^3^* | Asp; N *^4^* | Proportion of blood-feds (%) *^3^* | Farm; N *^1^* | Trap; N *^2^* | Proportion of blood-feds (%) *^3^* | Asp; N *^4^* | Proportion of blood-feds (%) *^3^* |
| Early June (June 6 – 7) | 3 | 6 | 0/1 (0) | 4 | 8 | 0/257 (0) | 8 | 0/7 (0) | 4 | 8 | 3/290 (1.0) | 8 | 5/41 (12.2) |
| Late June (June 20 – 21) | NA | NA | NA | 4 | 8 | 4/418 (1) | 8 | 4/62 (6.5) | 4 | 8 | 3/201 (1.5) | 8 | 3/114 (2.6) |
| Early July (July 4 – 5) | 4 | 8 | 29/577 (5.0) | 4 | 8 | 6/450 (1.3) | 8 | 3/41 (7.3) | 4 | 8 | 1/218 (0.5) | 8 | 8/110 (7.3) |
| Late July (July 18 – 19) | 4 | 8 | 0/226 (0) | 4 | 8 | 0/386 (0) | 8 | 0/223 (0) | 4 | 8 | 1/323 (0.3) | 8 | 13/669 (1.9) |
| Early August (August 1 – 2) | 4 | 8 | 4/457 (0.9) | 4 | 8 | 0/463 (0) | 8 | 3/254 (1.2) | 4 | 8 | 0/161 (0) | 8 | 11/177 (6.2) |
| Mid-August (August 15 – 16) | 4 | 8 | 1/158 (4.4) | 4 | 8 | 0/412 (0) | 8 | 2/205 (1.0) | 4 | 8 | 3/147 (2.0) | 8 | 1/83 (1.2) |
| Early September (September 5 – 6) | 4 | 8 | 0/74 (0) | 4 | 8 | 1 (0.5) | 8 | 1/246 (0.4) | 4 | 8 | 1/107 (0.9) | 8 | 1/165 (0.6) |
| Mid-September (September 12 – 13) | 4 | 8 | 1/174 (0.6) | 4 | 8 | 0/231 (0) | 8 | 0/204 (0) | 4 | 8 | 0/91 (0) | 8 | 0/173 (0) |
| Late September (September 26 – 27) | 4 | 8 | 1/134 (0.7) | 4 | 8 | 0/63 (0) | 8 | 0/78 (0) | 4 | 8 | 0/29 (0) | 8 | 0/56 (0) |
| Early October (October 10 – 11) | 4 | 8 | 0/52 (0) | 4 | 8 | 0/14 (0) | 8 | 0/15 (0) | 4 | 8 | 0/10 (0) | 8 | 0/8 (0) |
| Total |  |  | 36/1,853 (1.9) |  |  | 11/2,898 (0.4) |  | 13/1,336 (1.0) |  |  | 12/1,577 (0.8) |  | 42/1,596 (2.6) |

NA indicates that sampling was not conducted.

*^1^* Number of farms sampled during that calendar period.

*^2^* Number of traps set across the farms during each calendar period.

*^3^* Proportion of blood-fed mosquitoes was calculated as the number of blood-fed mosquitoes divided by the total number of female mosquitoes, multiplied by 100 to express the result as a percentage.

*^4^* Number of aspirations (Asp) conducted across farms during each calendar period.

**Supplementary Table 10**. The total proportion and percentage of blood-fed mosquitoes captured by BG traps and aspirators by calendar period and location (indoor/water body/vegetation) across **wean-to-market farms**.

|  | Indoor | | | Water bodies | | | | | Vegetation | | | | |
| --- | --- | --- | --- | --- | --- | --- | --- | --- | --- | --- | --- | --- | --- |
| Calendar period | Farm; N *^1^* | Trap; N *^2^* | Proportion of blood-feds (%) *^3^* | Farm; N *^1^* | Trap; N *^2^* | Proportion of blood-feds (%) *^3^* | Asp; N *^4^* | Proportion of blood-feds (%) *^3^* | Farm; N *^1^* | Trap; N *^2^* | Proportion of blood-feds (%) *^3^* | Asp; N *^4^* | Proportion of blood-feds (%) *^3^* |
| Early June (June 6 – 7) | 3 | 6 | 0/0 (0) | 4 | 8 | 0/50 (0) | 8 | 0/0 (0) | 4 | 8 | 0/1 (0) | 8 | 0/0 (0) |
| Late June (June 20 – 21) | NA | NA | NA | 4 | 8 | 0/46 (0) | 8 | 0/0 (0) | 4 | 8 | 0/41 (0) | 8 | 2/10 (20.0) |
| Early July (July 4 – 5) | 4 | 8 | 2/31 (6.5) | 4 | 8 | 0/278 (0) | 8 | 1/17 (5.9) | 4 | 8 | 0/413 (0) | 8 | 1/189 (0.5) |
| Late July (July 18 – 19) | 4 | 8 | 9/440 (2.0) | 4 | 8 | 0/328 (0) | 8 | 0/25 (0) | 4 | 8 | 0/307 (0) | 8 | 2/303 (0.7) |
| Early August (August 1 – 2) | 4 | 8 | 36/518 (6.9) | 4 | 8 | 5/521 (1.0) | 8 | 0/54 (0) | 4 | 8 | 3/200 (1.5) | 8 | 1/123 (0.8) |
| Mid-August (August 15 – 16) | 4 | 8 | 48/437 (11.0) | 4 | 8 | 0/77 (0) | 8 | 0/4 (0) | 4 | 8 | 2/140 (1.4) | 8 | 3/76 (3.9) |
| Early September (September 5 – 6) | 4 | 8 | 129/604 (21.4) | 4 | 8 | 0/75 (0) | 8 | 0/23 (0) | 4 | 8 | 1/81 (1.2) | 8 | 0/81 (0) |
| Mid-September (September 12 – 13) | 4 | 8 | 45/103 (43.7) | 4 | 8 | 1/75 (1.3) | 8 | 1/31 (3.2) | 4 | 8 | 2/77 (2.6) | 8 | 2/169 (1.2) |
| Late September (September 26 – 27) | 4 | 8 | 33/50 (66.0) | 4 | 8 | 0/9 (0) | 8 | 0/7 (0) | 4 | 8 | 0/16 (0) | 8 | 0/20 (0) |
| Early October (October 10 – 11) | 4 | 8 | 1/74 (1.4) | 4 | 8 | 0/12 (0) | 8 | 0/2 (0) | 4 | 8 | 0/11 (0) | 8 | 0/4 (0) |
| Total |  |  | 303/2,257 (13.4) |  |  | 6/1,471  (0.4) |  | 2/163  (1.2) |  |  | 8/1,287  (0.6) |  | 11/985  (1.1) |

NA indicates that sampling was not conducted.

*^1^* Number of farms sampled during that calendar period.

*^2^* Number of traps set across the farms during each calendar period.

*^3^* Proportion of blood-fed mosquitoes was calculated as the number of blood-fed mosquitoes divided by the total number of female mosquitoes, multiplied by 100 to express the result as a percentage.

*^4^* Number of aspirations (Asp) conducted across the farms during each calendar period.

**Supplementary Table 11**. Total number of host species identified and the proportion*^1^* of blood-fed mosquitoes feeding on each host species, stratified by location (indoors, near water bodies, and vegetation) and capture method (BG traps and aspirators) on **sow farms**.

| Species | Pigs | | | | | Cattle | | | | | White-tailed deer | | | | | Birds *^2^* | | | | | Total |
| --- | --- | --- | --- | --- | --- | --- | --- | --- | --- | --- | --- | --- | --- | --- | --- | --- | --- | --- | --- | --- | --- |
|  | TI | TW | TV | AW | AV | TI | TW | TV | AW | AV | TI | TW | TV | AW | AV | TI | TW | TV | AW | AV |  |
| *Ae. vexans* | 12/12 | 0/0 | 2/4 | 3/6 | 16/28 | 0/12 | 0/0 | 0/4 | 0/6 | 0/28 | 0/12 | 0/0 | 2/4 | 3/6 | 12/28 | 0/12 | 0/0 | 0/4 | 0/6 | 0/28 | 50 |
| *Cx. salinarius* | 21/21 | 3/4 | 1/3 | 0/2 | 2/2 | 0/21 | 0/4 | 1/3 | 0/2 | 0/2 | 0/21 | 1/4 | 1/3 | 0/2 | 0/2 | 0/21 | 0/4 | 0/3 | 2/2 | 0/2 | 32 |
| *Cx. pipiens* | 2/2 | 1/4 | 1/2 | 4/4 | 4/4 | 0/2 | 0/4 | 0/2 | 0/4 | 0/4 | 0/2 | 3/4 | 0/2 | 0/4 | 0/4 | 0/2 | 0/4 | 1/2 | 0/4 | 0/4 | 16 |
| *An. quadrimaculatus* | 1/1 | 0/2 | 0/2 | 0/0 | 4/4 | 0/1 | 0/2 | 0/2 | 0/0 | 0/4 | 0/1 | 2/2 | 2/2 | 0/0 | 0/4 | 0/1 | 0/2 | 0/2 | 0/0 | 0/4 | 9 |
| *Cx. erraticus* | 0/0 | 1/1 | 1/1 | 1/1 | 2/2 | 0/0 | 0/1 | 0/1 | 0/1 | 0/2 | 0/0 | 0/1 | 0/1 | 0/1 | 0/2 | 0/0 | 0/1 | 0/1 | 0/1 | 0/2 | 5 |
| *An. punctipennis* | 0/0 | 0/0 | 0/0 | 0/0 | 1/1 | 0/0 | 0/0 | 0/0 | 0/0 | 0/1 | 0/0 | 0/0 | 0/0 | 0/0 | 0/1 | 0/0 | 0/0 | 0/0 | 0/0 | 0/1 | 1 |
| *Cx. tarsalis* | 0/0 | 0/0 | 0/0 | 0/0 | 0/1 | 0/0 | 0/0 | 0/0 | 0/0 | 0/1 | 0/0 | 0/0 | 0/0 | 0/0 | 1/1 | 0/0 | 0/0 | 0/0 | 0/0 | 0/1 | 1 |
| Total | 36 | 5 | 5 | 8 | 29 | 0 | 0 | 1 | 0 | 0 | 0 | 6 | 5 | 3 | 13 | 0 | 0 | 1 | 2 | 0 | 114 |

Abbreviations: TI = Trap Indoors; TW = Trap Water body; TV = Trap Vegetation; AW = Aspirator Water body; AV = Aspirator Vegetation

*^1^* Proportion of blood-fed mosquitoes was calculated as the number of females of a given mosquito species, collected by a specific capture method and habitat, that fed on a particular host, divided by the total number of blood-fed females of that species captured by the same method within that habitat.

*^2^* For sow farms, Turkey Vulture [(*Cathartes aura*) identified from blood meals of *Cx. pipiens*] was the only bird host identified.

**Supplementary Table 12.** Total number of host species identified and the proportion*^1^* of blood-fed mosquitoes feeding on each host species, stratified by location (indoors, near water bodies, and vegetation) and capture method (BG traps and aspirators) on **wean-to-market farms**.

| Species | Domestic pigs | | | | | Domestic cattle | | | | | White-tailed deer | | | | | Birds *^2^* | | | | | Total |
| --- | --- | --- | --- | --- | --- | --- | --- | --- | --- | --- | --- | --- | --- | --- | --- | --- | --- | --- | --- | --- | --- |
|  | TI | TW | TV | AW | AV | TI | TW | TV | AW | AV | TI | TW | TV | AW | AV | TI | TW | TV | AW | AV |  |
| *An. quadrimaculatus* | 134/143 | 0/0 | 2/3 | 1/1 | 0/1 | 3/143 | 0/0 | 1/3 | 0/1 | 1/1 | 4/143 | 0/0 | 0/3 | 0/1 | 0/1 | 2/143 | 0/0 | 0/3 | 0/1 | 0/1 | 148 |
| *Cx. pipiens* | 77/81 | 2/2 | 2/2 | 0/0 | 2/3 | 2/81 | 0/2 | 0/2 | 0/0 | 0/3 | 0/81 | 0/2 | 0/2 | 0/0 | 0/3 | 2/81 | 0/2 | 0/2 | 0/0 | 1/3 | 88 |
| *An. punctipennis* | 40/42 | 0/1 | 1/1 | 0/0 | 0/0 | 0/42 | 0/1 | 0/1 | 0/0 | 0/0 | 2/42 | 0/1 | 1/1 | 0/0 | 0/0 | 0/42 | 0/1 | 0/1 | 0/0 | 0/0 | 44 |
| *Ae. vexans* | 10/10 | 0/0 | 0/0 | 0/1 | 3/4 | 0/10 | 0/0 | 0/0 | 0/1 | 0/4 | 0/10 | 0/0 | 0/0 | 1/1 | 1/4 | 0/10 | 0/0 | 0/0 | 0/1 | 0/4 | 15 |
| *Cx. salinarius* | 9/9 | 0/4 | 0/1 | 0/0 | 0/0 | 0/9 | 2/4 | 1/1 | 0/0 | 0/0 | 0/9 | 1/4 | 0/1 | 0/0 | 0/0 | 0/9 | 1/4 | 0/1 | 0/0 | 0/0 | 14 |
| *Cx. tarsalis* | 8/8 | 0/0 | 0/0 | 0/0 | 0/0 | 0/8 | 0/0 | 0/0 | 0/0 | 0/0 | 0/8 | 0/0 | 0/0 | 0/0 | 0/0 | 0/8 | 0/0 | 0/0 | 0/0 | 0/0 | 8 |
| *Ps. columbiae* | 8/8 | 0/0 | 0/0 | 0/0 | 0/0 | 0/8 | 0/0 | 0/0 | 0/0 | 0/0 | 0/8 | 0/0 | 0/0 | 0/0 | 0/0 | 0/8 | 0/0 | 0/0 | 0/0 | 0/0 | 8 |
| *Ae. japonicus* | 0/0 | 0/0 | 0/0 | 0/0 | 1/1 | 0/0 | 0/0 | 0/0 | 0/0 | 0/1 | 0/0 | 0/0 | 0/0 | 0/0 | 0/1 | 0/0 | 0/0 | 0/0 | 0/0 | 0/1 | 1 |
| *Cx. erraticus* | 1/1 | 0/0 | 0/0 | 0/0 | 0/0 | 0/1 | 0/0 | 0/0 | 0/0 | 0/0 | 0/1 | 0/0 | 0/0 | 0/0 | 0/0 | 0/1 | 0/0 | 0/0 | 0/0 | 0/0 | 1 |
| *Cx. restuans* | 0/0 | 0/0 | 0/0 | 0/0 | 0/1 | 0/0 | 0/0 | 0/0 | 0/0 | 0/1 | 0/0 | 0/0 | 0/0 | 0/0 | 0/1 | 0/0 | 0/0 | 0/0 | 0/0 | 1/1 | 1 |
| *Oc. dorsalis* | 0/0 | 0/0 | 0/0 | 0/0 | 0/1 | 0/0 | 0/0 | 0/0 | 0/0 | 0/1 | 0/0 | 0/0 | 0/0 | 0/0 | 1/1 | 0/0 | 0/0 | 0/0 | 0/0 | 0/1 | 1 |
| *Oc. trivittatus* | 1/1 | 0/0 | 0/0 | 0/0 | 0/0 | 0/1 | 0/0 | 0/0 | 0/0 | 0/0 | 0/1 | 0/0 | 0/0 | 0/0 | 0/0 | 0/1 | 0/0 | 0/0 | 0/0 | 0/0 | 1 |
| Total | 288 | 2 | 5 | 1 | 6 | 5 | 2 | 2 | 0 | 1 | 6 | 1 | 1 | 1 | 2 | 4 | 1 | 0 | 0 | 2 | 330 |

Abbreviations: TI = Trap Indoors; TW = Trap Water body; TV = Trap Vegetation; AW = Aspirator Water body; AV = Aspirator Vegetation

*^1^* Proportion of blood-fed mosquitoes was calculated as the number of females of a given mosquito species, collected by a specific capture method and habitat, that fed on a particular host, divided by the total number of blood-fed females of that species captured by the same method within that habitat.

*^2^* Bird species identified includes the Turkey Vulture [(*Cathartes aura*) identified from *An. quadrimaculatus, Cx. pipiens,* and *Cx. salinarius*], American robin [(*Turdus migratorius*) identified from *Cx. pipiens*), and Swainson’s thrush [(*Catharus ustulatus*) identified from *Cx. restuans*).
